# Factors Influence Breastfeeding Duration after High Risk and Low Risk Pregnancies

**DOI:** 10.3390/healthcare12181896

**Published:** 2024-09-21

**Authors:** Panagiota Brani, Maria Iliadou, Ewa Andersson, Georgios Daskalakis, Peter Drakakis, Maria Dagla

**Affiliations:** 1 Department of Midwifery, School of Health & Care Sciences, University of West Attica, 12243 Athens, Greece; pbrani@uniwa.gr (P.B.); miliad@uniwa.gr (M.I.); 2 Department of Women’s and Children’s Health, Division of Reproductive Health, Karolinska Institutet, 17177 Stockholm, Sweden; ewa.andersson@ki.se; 3 First Department of Obstetrics and Gynecology, Medical School, National and Kapodistrian University of Athens, General Hospital “ALEXANDRA”, 11528 Athens, Greece; gdaskalakis@med.uoa.gr; 4 Third Department of Obstetrics and Gynecology, Medical School, National and Kapodistrian University of Athens, University Hospital “ATTIKON”, 12461 Athens, Greece; pdrakakis@med.uoa.gr

**Keywords:** breastfeeding duration, high-risk pregnancy, exclusive breastfeeding, mixed feeding, predictive factors, birth weight, maternal health

## Abstract

Background: Breastfeeding provides vital nutrients and fosters maternal–infant bonding, benefiting both mother and child. However, breastfeeding duration is influenced by various factors, especially in women of high-risk pregnancy. This study aims to identify predictors of breastfeeding duration among women of high-risk and low-risk pregnancy, focusing on exclusive and mixed feeding practices. Methods: Conducted at a public hospital in Attica, Greece, this 20-month prospective cohort study (May 2020–January 2022) included 380 pregnant women, divided into high-risk and low-risk groups. The final sample of 157 women, after excluding non-breastfeeding participants, was assessed across five phases from prenatal to six months postpartum using interviews, calls, and surveys. Multiple linear regression identified key predictors, with statistical methods applied. Results: Results revealed birth weight as a consistent significant predictor of breastfeeding duration. For women with high-risk pregnancy, additional factors like infant gender, jaundice, and early introduction of solid foods influenced breastfeeding duration. The mixed breastfeeding model for women with high-risk pregnancy explained 72.9% of the variance. Exclusive and mixed breastfeeding models for women with low-risk pregnancy also highlighted birth weight’s influence. Conclusion: The findings highlight the important role of birth weight and other specific factors in determining breastfeeding duration among women of high-risk and low-risk pregnancy. Further research is needed to validate these findings across diverse populations.

## 1. Introduction

Breastfeeding is widely recognized for its significant health benefits for both infants and mothers, providing essential nutrients and fostering a strong maternal–infant bond [1,2,3,4]. The World Health Organization (WHO) recommends exclusive breastfeeding for the first six months of life, followed by continued breastfeeding along with appropriate complementary foods up to two years of age or beyond [4,5,6]. Despite these recommendations, breastfeeding practices vary widely, influenced by a multitude of factors including maternal health, socio-demographic characteristics, and healthcare practices [7,8,9,10].

Worldwide, 44% of infants under six months old are exclusively breastfed, with this proportion increasing to 68% for any form of breastfeeding, including both exclusive and mixed feeding, by the time they reach one year of, as stated in Global Breastfeeding Scorecard 2021 of the WHO [11]. An international target aims to elevate the prevalence of exclusive breastfeeding to 70% for infants up to six months of age and to achieve an 80% rate for breastfeeding either exclusive or not by the end of the first year by 2030 [12]. The WHO European Region exhibits the lowest global rates of exclusive breastfeeding at the six-month milestone, with only 25% of infants exclusively breastfed at this age. In contrast, in the WHO African Region, the rate is much higher, reaching approximately 43% [12,13].

The duration of breastfeeding maintains an important role in determining health outcomes for both the mother and the infant [5,14,15,16]. Nonetheless, adhering to the recommended breastfeeding duration poses significant challenges, especially for women experiencing high-risk pregnancy, which is defined as a pregnancy with increased health risks for the mother or baby due to factors such as maternal age, pre-existing medical conditions, complications during pregnancy, or multiple gestations [8,17,18]. Conditions such as gestational diabetes, hypertension, and other medical complications can impede a mother’s capacity to commence and continue breastfeeding [19,20,21,22,23]. Conversely, gaining insights into how these conditions influence breastfeeding practices, either exclusive or not, can guide the development of targeted interventions designed to support these women and enhance breastfeeding outcomes [24,25,26].

This study aims to identify the predictive factors influencing the duration of breastfeeding among women of high-risk and low-risk pregnancy. Specifically, it compares breastfeeding duration in women who either exclusively breastfeed their infants or engage in mixed feeding practices, where breast milk is supplemented with other liquid foods [11]. By examining these distinct groups, the research seeks to elucidate how various maternal and infant characteristics, including the presence of high-risk pregnancy condition, affect breastfeeding behaviors and outcomes. This study aims to provide an understanding of the factors that influence breastfeeding duration.

## 2. Materials and Methods

### 2.1. Study Design and Objectives

This prospective cohort study was designed to identify and analyze the predictive factors influencing the duration of breastfeeding among women with high-risk pregnancy compared to those with low-risk pregnancy. The study focused on two specific breastfeeding practices: exclusive breastfeeding and mixed breastfeeding, where infants receive both breast milk and other liquid foods. By comparing these groups, the research aimed to provide a comprehensive understanding of how various maternal and infant characteristics, particularly the presence of high-risk pregnancy, impact breastfeeding duration. Exclusive breastfeeding is defined as feeding the infant only breast milk, without any additional liquids or solids, except for oral rehydration salts, vitamins, or medications, until six months of age. Mixed feeding refers to the practice of supplementing breast milk with other liquids, such as water or formula, starting after six months [27].

### 2.2. Setting and Participants

The study was conducted over a 20-month period, from May 2020 to January 2022, at a public hospital located in Attica, Greece. It is important to note a significant reduction in births from 2020 to 2023, with the number of births decreasing from 4.250 in 2020 to 2.937 in 2023. Additionally, the rate of cesarean sections is at 62%, with the majority of births involving women hospitalized due to pregnancy complications, according to the hospital records. The study’s participants were selected based on specific eligibility criteria to ensure a representative sample. Eligible participants included women who were at least 18 years of age, fluent in the Greek language, and who had delivered their infants at the hospital with a gestational age of 32 weeks or more. These criteria were established to ensure that the participants had a sufficient level of language comprehension and that the infants were of a viable gestational age for breastfeeding studies. Women were included in the study as having high-risk pregnancy if they had a prenatal hospitalization in the high-risk pregnancy clinic for more than 48 h due to one or more of the following conditions: Gestational Diabetes Mellitus (GDM), which requires medical management to control blood glucose levels; Gestational Hypertension, Pre-eclampsia, or Eclampsia, characterized by high blood pressure during pregnancy with potential complications for both mother and infant; Fetal Growth Restriction (FGR), where the fetus is not growing at the expected rate inside the womb; Small for Gestational Age (SGA), indicating that the infant’s weight is below the 10th percentile for gestational age; risk of preterm labor; vaginal bleeding; placental disorders; and systemic diseases that pre-existed pregnancy, such as chronic hypertension and Type I or Type II diabetes mellitus.

### 2.3. Participant Recruitment and Sample Size

The initial dataset comprised 380 pregnant women, stratified into two categories based on their risk status: 200 women were identified as high-risk, while 180 were classified as low-risk. The methodology used for sampling, along with the recorded response rates of 82% for the high-risk group and 85% for the low-risk group, was carefully documented. The sample comprised 164 women with high-risk pregnancies and 154 women with low-risk pregnancies. To refine the sample for this study, women who did not engage in breastfeeding or who exclusively fed their infants formula were excluded. This filtering process resulted in a final sample of 157 women, which included 59 high-risk pregnancies and 98 low-risk pregnancies.

### 2.4. Data Collection

Data collection was organized into five distinct phases, spanning from the prenatal period to six months postpartum. A mixed-methods approach was employed, incorporating in-person interviews, telephone calls, and online surveys conducted via Google Forms. The phases were:

Phase 1: Prenatal data collection was conducted during hospitalization and outpatient visits. This phase involved administering detailed questionnaires to capture socio-demographic information, medical history, and obstetric characteristics of the participants.

Phase 2: Data collection occurred on the 3rd to 4th day postpartum, focusing on the initial feeding methods employed by the mothers. Information gathered during this phase included the type of breastfeeding practiced (exclusive or mixed), any supplementary feeding given, and the early breastfeeding experiences of the participants.

Phase 3: At the end of the puerperium period, assessments were carried out through phone interviews or online questionnaires. This phase aimed to evaluate breastfeeding outcomes, documenting any changes in feeding practices and the factors influencing these changes.

Phase 4: Data collection at three months postpartum involved follow-up phone interviews or electronic forms. This phase assessed the ongoing breastfeeding status of the participants, capturing data on the continuation of breastfeeding, any introduction of supplementary foods, and the overall breastfeeding experience up to that point.

Phase 5: The final phase of data collection took place at six months postpartum. This phase aimed to evaluate long-term breastfeeding practices, including the duration of exclusive or mixed breastfeeding, the factors contributing to continued breastfeeding, and any challenges faced by the mothers.

### 2.5. Research Instruments

To achieve the study’s objectives, the following self-reported questionnaires were constructed and administered to the participants at corresponding phases:

▪ Q-1: Socioeconomic, demographic, and obstetric characteristics questionnaire (completed during pregnancy, after 32 weeks).
▪ Q-2: Postpartum questionnaire from birth to the 3rd-4th day after birth (includes birth details, neonatal characteristics, type of feeding in the hospital, etc.).
▪ Q-3: Breastfeeding outcome questionnaire at the end of the puerperium.
▪ Q-4: Breastfeeding outcome questionnaire at the end of the 3rd month.
▪ Q-5: Breastfeeding outcome questionnaire at the end of the 6th month.

### 2.6. Ethical Considerations

This study was conducted following the ethical standards outlined in the Declaration of Helsinki and received approval from the Institutional Review Board (IRB) of the public hospital under protocol number 346, dated 20 May 2020. All participants were informed about the study’s objectives, methodology, potential risks, and benefits, and provided written informed consent. Participant data was anonymized and handled with secure storage protocols to ensure confidentiality.

### 2.7. Data Analysis

**Figure 1:**
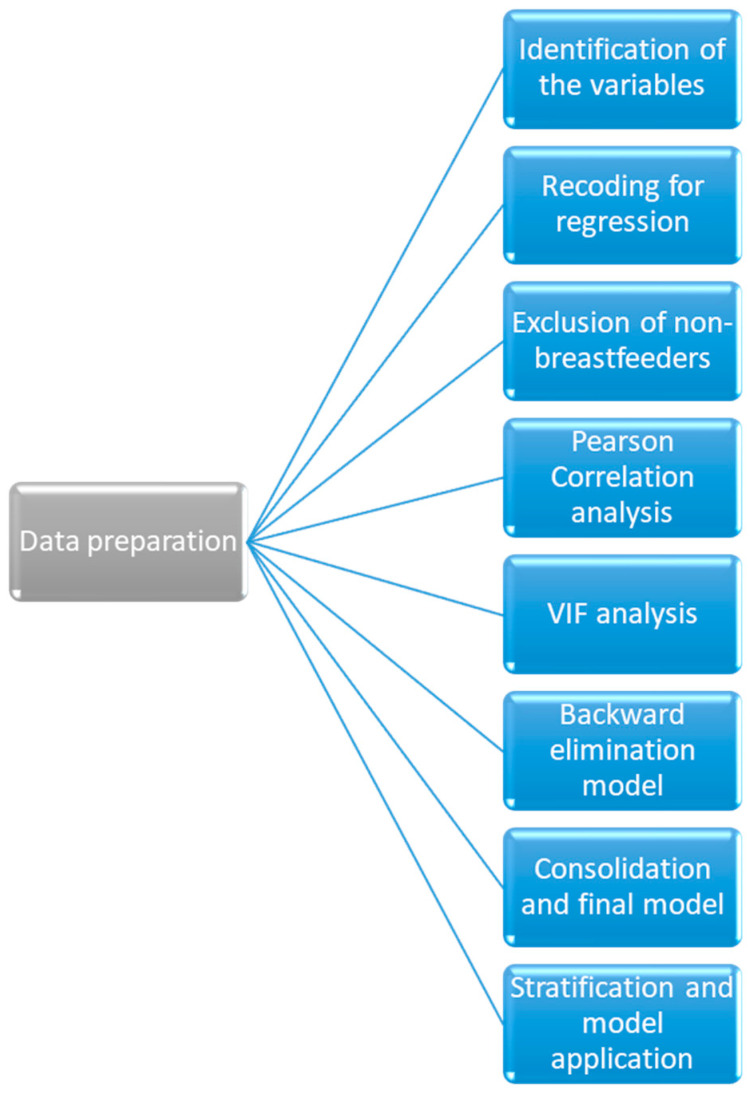
Data preparation and analysis process.

A statistical analysis was executed employing the Statistical Package for the Social Sciences (SPSS), version 22.0. The data preparation and analysis process in this study was conducted with attention to detail to ensure the reliability of the findings. The following steps outline the comprehensive approach taken, with justifications provided for each methodological decision (Figure 1).

- Identification of Relevant Variables

The initial step involved identifying the variables pertinent to the infant and the hospital. This included socio-demographic characteristics, medical history, obstetric factors, and hospital-related details. These variables were selected based on their potential influence on breastfeeding duration, as suggested by existing literature and clinical expertise. In our research, the following variables were identified and collected: maternal age, gestational age, birth weight, infant gender, presence of neonatal jaundice, mode of delivery, and feeding method (exclusive or mixed breastfeeding). These variables were selected based on their potential influence on breastfeeding duration, as indicated by the existing literature. From these, the variables that were statistically evaluated using multiple linear regression models included birth weight, infant gender, gestational age, and the presence of neonatal jaundice. These variables were analyzed to determine their significance in predicting the duration of breastfeeding among high-risk and low-risk pregnancies.

- Recoding Variables for Regression Models

To facilitate the inclusion of identified variables in regression models, necessary recoding was performed. This step involved converting categorical variables into dummy variables and ensuring all variables were appropriately formatted for analysis. For example, categorical variables such as type of delivery, the presence of a high-risk pregnancy condition, and initial feeding methods were recoded into binary or ordinal scales as required for regression analysis.

- Exclusion of Non-Breastfeeding Participants

Women who exclusively fed their children formula or did not breastfeed at all were excluded from the analysis. This exclusion was justified to focus the study on breastfeeding behaviors and ensure that the dependent variable, breastfeeding duration, was relevant and accurately measured across the sample. This filtering process resulted in a final sample of 157 women, all of whom engaged in either exclusive or mixed breastfeeding.

- Pearson Correlation Analysis

A Pearson correlation analysis was employed to identify variables with the highest correlations with the dependent variable, breastfeeding duration. Variables with significant correlations were selected for further analysis, ensuring that the models included only the most relevant predictors.

- Variance Inflation Factor (*VIF*) Analysis

To address the issue of multicollinearity among independent variables, a Variance Inflation Factor (*VIF*) analysis was conducted. *VIF* values were calculated for all predictor variables, and those exhibiting high *VIF* values (indicative of multicollinearity) were removed from the model. *VIF* analysis is a diagnostic tool used to detect multicollinearity among predictor variables in a regression model. Multicollinearity occurs when two or more predictor variables in a model are highly correlated, which can lead to inflated standard errors and unreliable coefficient estimates. *VIF* analysis quantifies how much the variance of a regression coefficient is inflated due to multicollinearity [26,27].

The process of *VIF* analysis begins with constructing the full regression model. This involves fitting a multiple regression model that includes all the predictor variables of interest. By starting with a comprehensive model, the analysis ensures that all potential relationships among the variables are considered. Once the full regression model is established, the next step is to calculate the *VIF* for each predictor variable. This calculation involves determining how much the variance of each regression coefficient is inflated due to the presence of multicollinearity. The *VIF* for a predictor variable is computed using the following formula:

$$VIFi=\frac{1}{1-R_{i}^{2}}$$

where $R_{i}^{2}$ is the coefficient of determination obtained by regressing the *i*-th predictor on all other predictors in the model. To detect and address multicollinearity among predictor variables, a series of auxiliary regressions were conducted, wherein each predictor was treated as the dependent variable in turn, with all other predictors serving as independent variables. The subsequent step involved interpreting the *VIF* values. *VIF* provides a quantitative measure of the extent to which multicollinearity inflates the variance of a regression coefficient. A *VIF* value of 1 indicates no multicollinearity, values between 1 and 5 suggest moderate multicollinearity, values above 5 indicate high multicollinearity, and values exceeding 10 are considered very high, signaling severe multicollinearity issues. When *VIF* values exceed 10, indicating significant multicollinearity, corrective actions are necessary. One approach involves removing the variables with the highest *VIF* values and re-evaluating the model. Alternatively, highly correlated variables can be combined into a single composite variable to reduce multicollinearity. Additionally, techniques such as Principal Component Analysis (PCA) or ridge regression can be employed to manage multicollinearity effectively. Following the *VIF* analysis and the implementation of appropriate adjustments, the revised regression model is re-evaluated to ensure that multicollinearity has been sufficiently addressed and that the model provides reliable and interpretable coefficient estimates.

- Application of Backward Elimination Method

A backward elimination method was applied to each group of independent variables, categorized based on the time of data collection. This iterative process involved fitting the regression model and sequentially removing the least significant variables until only those with substantial predictive power remained. This approach helped to refine the model and exclude variables that did not contribute meaningfully to explaining breastfeeding duration. The backward elimination method is a stepwise regression technique used to refine statistical models by systematically removing non-significant variables. This ensures that the final model includes only the most relevant predictors, enhancing both interpretability and predictive accuracy [28,29].

In this study, backward elimination was employed for several reasons. Firstly, it aids in model simplification by starting with a full model and systematically removing the least significant variables. This reduces the risk of overfitting and makes the model more generalizable. Secondly, backward elimination enhances interpretability by focusing on the most impactful variables, thereby clarifying the relationship between predictors and the outcome variable—breastfeeding duration. Moreover, backward elimination helps identify the most critical predictors, ensuring that the final model includes only those variables that significantly impact breastfeeding duration. Lastly, backward elimination assists in managing multicollinearity by removing variables that do not significantly contribute to the model, thereby reducing multicollinearity among predictors. This leads to more stable and reliable coefficient estimates, enhancing the overall reliability of the model.

- Consolidation and Final Model Fitting

The final variables retained from each group were then consolidated, and a new regression model was fitted. This step involved further elimination of non-significant variables to arrive at the most parsimonious model. The consolidation ensured that the final model included only those variables that consistently demonstrated significant predictive power across different phases of data collection.

- Stratification and Model Application

The sample was stratified into four distinct groups based on the presence or absence of a high-risk pregnancy condition and whether the women practiced exclusive or mixed breastfeeding. This stratification was justified to explore how these different conditions affected breastfeeding duration. Four separate regression models were then applied, one for each stratum, allowing for a detailed analysis of the predictors within each specific context.

## 3. Results

The sample characteristics of the study are outlined in Table 1. Among the participants, 123 women exclusively breastfed their infants, which constitutes 78.3% of the sample, while 34 women (21.7%) practiced mixed breastfeeding. Regarding the presence of high-risk pregnancy, 98 women (62.4%) did not report any high-risk pregnancy conditions, whereas 37.6% (*n* = 59) had a high-risk pregnancy. Furthermore, 57.3% of the women (*n* = 90) continued breastfeeding for more than 40 days, compared to 42.7% (*n* = 67) who ceased breastfeeding within 40 days.

The crosstabulation between groups (high-risk pregnancy and low-risk pregnancy) and breastfeeding duration (up to 40 days and more than 40 days) provides significant data on the distribution and relationship of these variables within the sample. Among the 59 women with high-risk pregnancy, 64.4% (*n* = 38) ceased breastfeeding within 40 days, while 35.6% (*n* = 21) continued for more than 40 days. In contrast, among the 98 women with low-risk pregnancy, 29.6% (*n* = 29) ceased breastfeeding within 40 days, whereas a substantial 70.4% (*n* = 69) breastfed for more than 40 days. This indicates a significant difference in breastfeeding duration based on the presence of a high-risk pregnancy.

The overall sample consists of 157 participants, with 42.7% (*n* = 67) breastfeeding for up to 40 days and 57.3% (*n* = 90) breastfeeding for more than 40 days. The data suggests a significant association between the presence of a high-risk pregnancy and the duration of breastfeeding. Women with high-risk pregnancy are more likely to cease breastfeeding within the first 40 days compared to women with low-risk pregnancy, who predominantly continue breastfeeding beyond 40 days. This finding highlights the potential impact of high-risk pregnancy conditions on the ability to sustain breastfeeding for extended periods. The crosstabulation also reveals that the majority of women who breastfeed beyond 40 days are those of low-risk pregnancy. This indicates that a low-risk pregnancy favors longer breastfeeding durations, which may be associated with better maternal and infant health outcomes (Table 2).

The study applied four multiple linear regression models to investigate the factors influencing the duration of breastfeeding. The dependent variable in these models was the duration of breastfeeding, while the independent variables included a set of nine factors related to both the infant’s details and the hospital where the women gave birth. These nine independent variables were selected based on their higher linear correlation with the dependent variable and the absence of multicollinearity issues. Additionally, a backward elimination method was applied to ensure the final models contained only the most significant variables.

Model 1: Exclusive breastfeeding with high-risk pregnancy

The first model focused on women who practiced exclusive breastfeeding and had a high-risk pregnancy. This model underwent eight iterations, resulting in a final *R*-squared value of 0.202. This indicates that approximately 20.2% of the variance in breastfeeding duration for this group can be explained by the independent variables included in the model. The model was found to fit the data in a statistically significant manner, as evidenced by the ANOVA results (*F* (2, 41) = 5.177, *p* < 0.05) (Table 3).

The ANOVA table confirms the statistical significance of the model, with a *p*-value of 0.010, indicating that the model’s predictors collectively contribute to explaining the variation in breastfeeding duration (Table 4).

The coefficients table provides detailed insights into the impact of each independent variable on the duration of breastfeeding. For birth weight, the unstandardized coefficient is 0.000 with a *p*-value of 0.027, indicating that birth weight has a statistically significant positive effect on the duration of exclusive breastfeeding among women with high-risk pregnancy. Additionally, the unstandardized coefficient for infant’s gender is −0.286 with a *p*-value of 0.044, suggesting a statistically significant negative impact of infant gender on breastfeeding duration (Table 5). This means that, in the context of this study, higher birth weight is associated with longer durations of exclusive breastfeeding, while male gender of the infant is associated with shorter breastfeeding durations among female regarding the high-risk pregnancy conditions of the mother.

Model 2: Breastfeeding duration among women practicing exclusive breastfeeding with low-risk pregnancy

Continuing with the analysis of multiple linear regression models, the study focused on the model concerning women who practice exclusive breastfeeding but do not have a high-risk pregnancy. This model was refined through six iterations and resulted in an *R*-squared value of 0.276. This indicates that 27.6% of the variance in breastfeeding duration for this group can be explained by the included independent variables. The model demonstrates a statistically significant fit to the data, as confirmed by the ANOVA results (*F* (3, 74) = 9.387, *p* < 0.001) (Table 6).

The ANOVA table indicates that the model is statistically significant, with a *p*-value less than 0.001, which implies that the independent variables collectively contribute to explaining the variation in breastfeeding duration (Table 7).

The coefficients table provides detailed insights into the impact of each independent variable on the duration of breastfeeding. For birth weight, the unstandardized coefficient is 0.000 with a highly significant *p*-value (*p* < 0.001), indicating that birth weight is a significant predictor of breastfeeding duration among women of low-risk pregnancy. Specifically, higher birth weight is associated with longer breastfeeding durations. Despite the 0.000 coefficient suggesting a small effect size, its statistical significance highlights that even minor increases in birth weight can contribute to extended breastfeeding periods. For infant’s gender, the unstandardized coefficient is −0.182 with a *p*-value of 0.053, which is marginally above the conventional significance threshold of 0.05. This suggests a potential negative impact of male gender of the infant on breastfeeding duration, although this effect is not statistically significant at the 0.05 level. The variable representing how long the infant was breastfed has an unstandardized coefficient of 0.276 with a *p*-value of 0.057. This indicates a positive association with breastfeeding duration, but, like infant’s gender, it does not reach statistical significance at the 0.05 level (Table 8).

Model 3: Mixed breastfeeding with high-risk pregnancy

The third model focuses on women who practice mixed breastfeeding and have a high-risk pregnancy. This model underwent seven iterations, resulting in an exceptionally high R-squared value of 0.729, indicating that 72.9% of the variance in breastfeeding duration for this group can be explained by the included independent variables. The model fit the data in a statistically significant manner, as demonstrated by the ANOVA results (*F* (3, 10) = 8.953, *p* < 0.05) (Table 9).

The ANOVA table confirms the statistical significance of the model, with a *p*-value of 0.003, indicating that the independent variables collectively contribute significantly to explaining the variation in breastfeeding duration (Table 10).

The coefficients table provides insights into the impact of each independent variable on the duration of breastfeeding among women with high-risk pregnancy who practice mixed breastfeeding. For birth weight, the unstandardized coefficient is 0.000 with a *p*-value of 0.023, indicating that birth weight is a statistically significant predictor of breastfeeding duration. Higher birth weight is associated with longer breastfeeding duration, suggesting that infants with higher birth weights may have better health and feeding capacities, enabling prolonged breastfeeding. Regarding jaundice, the unstandardized coefficient is −0.535 with a *p*-value of 0.010, indicating a statistically significant negative impact on breastfeeding duration. Infants who presented with jaundice had shorter breastfeeding durations, likely due to health complications or feeding difficulties associated with the condition, which can hinder prolonged breastfeeding. Lastly, the beginning of solid foods has an unstandardized coefficient of 0.623 with a *p*-value of 0.021, suggesting a statistically significant positive impact on breastfeeding duration. Infants who started receiving solid foods up to the sixth month had longer breastfeeding durations. Introducing solid foods might support continued breastfeeding by complementing the infant’s diet, thereby sustaining breastfeeding practices for a longer period (Table 11).

Model 4: Mixed breastfeeding with low-risk pregnancy

The final model examines women who practice mixed breastfeeding and do not have a high-risk pregnancy. This model underwent eight iterations and resulted in an *R*-squared value of 0.348, indicating that 34.8% of the variance in breastfeeding duration for this group can be explained by the included independent variables. The model was found to fit the data in a statistically significant manner, as indicated by the ANOVA results (*F* (1, 16) = 8.539, *p* < 0.05) (Table 12). 

The ANOVA table confirms the statistical significance of the model, with a *p*-value of 0.010, indicating that the independent variables contribute significantly to explaining the variation in breastfeeding duration (Table 13).

The coefficients table provides insights into the impact of the independent variable on the duration of breastfeeding. For birth weight, the unstandardized coefficient is 0.001 with a *p*-value of 0.010, indicating that birth weight is a statistically significant predictor of breastfeeding duration among women who practice mixed breastfeeding with low-risk pregnancy. Specifically, higher birth weights are associated with longer breastfeeding durations (Table 14). This finding is consistent with previous models, highlighting the significant role that birth weight plays in sustaining breastfeeding practices. Higher birth weights may reflect better infant health and feeding capacities, enabling prolonged breastfeeding durations.

## 4. Discussion

The four multiple linear regression models presented in this study offer valuable information into the factors influencing the duration of breastfeeding among different groups of women. Each model was designed to assess the impact of various infant-related and maternal factors, taking into account whether the mothers practiced exclusive or mixed breastfeeding and whether they had a high-risk or a low-risk pregnancy.

The first model, which focuses on women practicing exclusive breastfeeding with a high-risk pregnancy, explains 20.2% of the variance in breastfeeding duration. This model is statistically significant, with birth weight and infant’s gender identified as significant predictors. Specifically, birth weight positively influences breastfeeding duration, indicating that higher birth weights are associated with longer breastfeeding periods. Conversely, the infant’s male gender is associated with a shorter duration of breastfeeding. The second model, which focuses on women practicing exclusive breastfeeding with a low-risk pregnancy, explains 27.6% of the variance in breastfeeding duration. This model is also statistically significant, with birth weight emerging as a significant predictor of breastfeeding duration. Higher birth weights are consistently associated with longer breastfeeding durations, emphasizing the critical role of infant weight in influencing breastfeeding practices. Although infant’s gender and the duration of previous breastfeeding show potential associations, their effects do not have statistical significance at the 0.05 level.

The third model, which examines women practicing mixed breastfeeding with a high-risk pregnancy, explains a substantial 72.9% of the variance in breastfeeding duration, making it a highly predictive model. This model is statistically significant, with birth weight, the presence of jaundice, and the introduction of solid foods emerging as significant predictors. Higher birth weights are associated with longer breastfeeding durations, while infants who presented with jaundice tend to have shorter durations. Additionally, the introduction of solid foods up to the sixth month is linked to extended breastfeeding duration. The fourth model, which focuses on women practicing mixed breastfeeding with a low-risk pregnancy, explains 34.8% of the variance in breastfeeding duration. This model is statistically significant, with birth weight emerging as the sole significant predictor. Higher birth weights are consistently associated with longer breastfeeding durations, indicating that infants with higher birth weights tend to breastfeed for extended periods.

The comparative analysis of these four models reveals several aspects. Firstly, birth weight consistently emerged as a significant predictor across all models, highlighting its role in determining breastfeeding duration. This finding suggests that higher birth weights are generally associated with longer breastfeeding periods, regardless of the breastfeeding practice or the presence of a high-risk pregnancy. Secondly, the presence of a high-risk pregnancy introduces additional complexity to breastfeeding behaviors. In women with a high-risk pregnancy, other factors such as infant’s gender and health indicators like jaundice significantly influence breastfeeding duration. Thirdly, the model for mixed breastfeeding with a high-risk pregnancy demonstrated the highest predictive power, explaining 72.9% of the variance in breastfeeding duration. This model’s high R-squared value underscores the importance of considering multiple factors, including birth weight, jaundice, and the introduction of solid foods, when addressing breastfeeding practices in women with high-risk pregnancies. In contrast, the models for exclusive breastfeeding with a low-risk pregnancy and mixed breastfeeding with a low-risk pregnancy had lower R-squared values but still provided valuable information. These models emphasize the consistent influence of birth weight on breastfeeding duration and suggest that other factors, while potentially influential, may not be as significant in the absence of a high-risk pregnancy condition.

**Figure 2:**
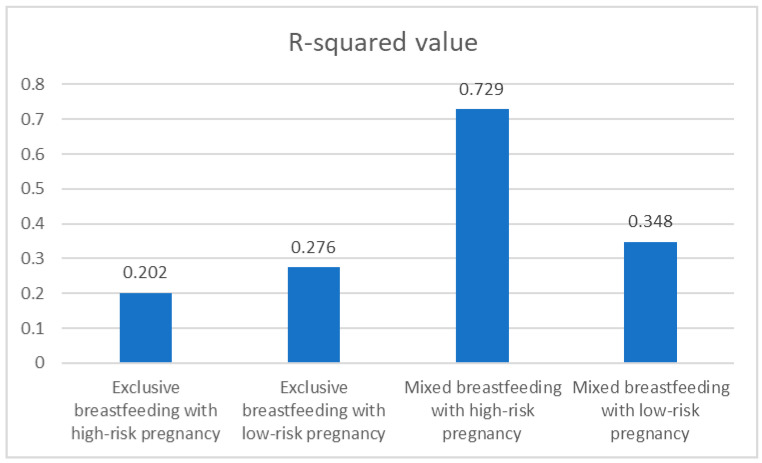
*R*-squared values of breastfeeding duration.

Figure 2 displays the R-squared values for each model, indicating the proportion of variance in breastfeeding duration explained by each model. The model for mixed breastfeeding with high-risk pregnancy has the highest R-squared value (0.729), followed by the models for mixed breastfeeding with low-risk pregnancy (0.348), exclusive breastfeeding with low-risk pregnancy (0.276), and exclusive breastfeeding with high-risk pregnancy (0.202).

The consistent identification of birth weight as a significant predictor across all models in this study aligns with findings from several existing studies. For instance, the study by Li et al. [30] found that higher birth weights were associated with longer breastfeeding durations, emphasizing the importance of infant weight in breastfeeding practices. Similarly, a study by Oddy et al. [31] reported that lower birth weight infants were at higher risk for early cessation of breastfeeding.

The influence of high-risk pregnancy conditions, such as jaundice, on breastfeeding duration is corroborated by previous research. While neonatal jaundice is a common medical complication that occurs after birth and affects the newborn, it should not be considered a pregnancy risk condition. In this study, neonatal jaundice was examined as a postnatal factor that may influence breastfeeding duration in high-risk pregnancies. For example, the study by Battersby et al. [32] found that neonatal jaundice was associated with shorter breastfeeding durations due to the potential feeding difficulties and maternal anxiety associated with the condition. The current study’s finding that the presence of a high-risk pregnancy introduces additional complexity to breastfeeding behaviors is consistent with the literature, suggesting the need for targeted interventions for mothers and infants experiencing health issues.

Moreover, the distinction between exclusive and mixed breastfeeding practices is well-documented in the literature. The current study’s finding that birth weight is a significant predictor for both exclusive and mixed breastfeeding practices a low-risk pregnancy is supported by a study by Grummer-Strawn et al. [33], which found that exclusive breastfeeding is often influenced by infant weight and maternal health. Additionally, the study by Scott et al. [34] reported that mixed breastfeeding practices were influenced by a combination of infant health indicators and maternal feeding decisions. While the current study found infant gender to be a significant predictor in the exclusive breastfeeding with a high-risk pregnancy model, some studies have reported mixed results regarding the influence of gender. For example, a study by Heck et al. [35] found no significant difference in breastfeeding duration based on infant gender. These discrepancies could be due to cultural, socioeconomic, or sample-specific differences, suggesting the need for further research to explore the contextual factors influencing gender-related breastfeeding behaviors.

This study has several limitations. The final analysis, involving 157 participants from a single public hospital in Attica, Greece, may limit the generalizability of the findings to other populations and settings. Reliance on self-reported data introduces potential recall and social desirability biases, affecting data accuracy. Although statistical methods were employed, some confounding variables like maternal mental health, support systems, and socio-economic status were not fully controlled. The heterogeneity within the high-risk group regarding the severity and management of conditions and the relatively short six-month follow-up period limit the understanding of long-term breastfeeding challenges. Variations in data collection methods may affect consistency, and excluding non-breastfeeding women limits insights into barriers to breastfeeding initiation. Finally, conducting the study at a single hospital may introduce institutional biases. Future research should address these limitations with larger, multi-center studies, longer follow-up periods, comprehensive control for confounders, and inclusion of non-breastfeeding women to enhance the generalizability of the findings.

The findings from these four multiple linear regression models have significant implications for both clinical practice and future research, providing a basis for developing tailored interventions, focusing on factors such as birth weight, and exploring comprehensive support programs. Future research should explore additional variables and potential interactions to enhance the explanatory power of these models. Larger and more diverse samples can help validate and extend these findings, providing a deeper understanding of the determinants of breastfeeding duration. Research could investigate the role of psychosocial factors, such as maternal stress and social support, the impact of different healthcare settings, and the influence of cultural practices on breastfeeding behaviors. By broadening the scope of research, we can develop a more comprehensive understanding of the various factors that affect breastfeeding duration and design more effective interventions to support breastfeeding mothers.

## 5. Conclusions

In summary, the four multiple linear regression models provide a comprehensive understanding of the factors influencing breastfeeding duration among different groups of women. The significant predictors identified across the models—birth weight, infant’s gender, the presence of jaundice, and the introduction of solid foods—underscore the multifaceted nature of breastfeeding practices. The findings of this study highlight the importance of early intervention and support for women with high-risk pregnancies to extend breastfeeding duration. This knowledge can help inform the development of targeted clinical protocols and educational programs, ultimately improving outcomes for both mothers and infants. Additionally, these results pave the way for future research to explore other factors not covered in this study, such as psychological support and social determinants.

## Figures and Tables

**Table 1:** Sample’s characteristics.

		Frequency (*n*)	Percentage (%)
Breastfeeding	Exclusive breastfeeding	123	78.3
	Mixed breastfeeding	34	21.7
Groups	High-risk	59	37.6
	Low-risk	98	62.4
Breastfeeding duration	Up to 40 days	67	42.7
	More than 40 days	90	57.3

**Table 2:** Crosstabulation between women with high-risk and low-risk pregnancy and breastfeeding duration.

		Breastfeeding Duration		Total
		Up to 40 Days	More Than 40 Days	
High-risk pregnancy	Frequency	38	21	59
	% within Groups	64.4%	35.6%	100.0%
	% of Total	24.2%	13.4%	37.6%
Low-risk pregnancy	Frequency	29	69	98
	% within Groups	29.6%	70.4%	100.0%
	% of Total	18.5%	43.9%	62.4%
Total	Frequency	67	90	157
	% within Groups	42.7%	57.3%	100.0%
	% of Total	42.7%	57.3%	100.0%

**Table 3:** Model summary (exclusive breastfeeding with high-risk pregnancy).

Model	*R*	*R* Square	Adjusted *R* Square	Std. Error of the Estimate
8	0.449	0.202	0.163	0.45071

**Table 4:** ANOVA (exclusive breastfeeding with high-risk pregnancy).

Model		Sum of Squares	*df*	Mean Square	*F*	Sig.
8	Regression	2.103	2	1.052	5.177	0.010
	Residual	8.329	41	0.203		
	Total	10.432	43			

**Table 5:** Coefficients (exclusive breastfeeding with high-risk pregnancy).

Model		Unstandardized Coefficients		Standardized Coefficients	*t*	Sig.
		*B*	Std. Error	Beta		
8	(Constant)	0.824	0.485		1.700	0.097
	Birth weight	0.000	0.000	0.321	2.294	0.027
	Infant’s gender	−0.286	0.138	−0.291	−2.076	0.044

**Table 6:** Model summary (exclusive breastfeeding with low-risk pregnancy).

Model	*R*	*R* Square	Adjusted *R* Square	Std. Error of the Estimate
6	0.525	0.276	0.246	0.38154

**Table 7:** ANOVA (exclusive breastfeeding with low-risk pregnancy).

Model		Sum of Squares	*df*	Mean Square	*F*	Sig.
6	Regression	4.099	3	1.366	9.387	0.000
	Residual	10.772	74	0.146		
	Total	14.872	77			

**Table 8:** Coefficients (exclusive breastfeeding with low-risk pregnancy).

Model		Unstandardized Coefficients		Standardized Coefficients	*t*	Sig.
		*B*	Std. Error	Beta		
6	(Constant)	−0.167	0.537		−0.310	0.757
	Birth weight	0.000	0.000	0.404	3.970	0.000
	Infant’s gender	−0.182	0.093	−0.200	−1.967	0.053
	How long did you breastfeed?	0.276	0.142	0.192	1.936	0.057

**Table 9:** Model summary (mixed breastfeeding with high-risk pregnancy).

Model	*R*	*R* Square	Adjusted *R* Square	Std. Error of the Estimate
7	0.854	0.729	0.647	0.27841

**Table 10:** ANOVA (mixed breastfeeding with high-risk pregnancy).

Model		Sum of Squares	*df*	Mean Square	*F*	Sig.
7	Regression	2.082	3	0.694	8.953	0.003
	Residual	0.775	10	0.078		
	Total	2.857	13			

**Table 11:** Coefficients (mixed breastfeeding with high-risk pregnancy).

Model		Unstandardized Coefficients		Standardized Coefficients	*t*	Sig.
		*B*	Std. Error	Beta		
7	(Constant)	−0.285	0.518		−0.551	0.594
	Birth weight	0.000	0.000	0.462	2.684	0.023
	Did your infant present with jaundice (increased bilirubin)?	−0.535	0.168	−0.586	−3.186	0.010
	Did you start giving solid foods?	0.623	0.228	0.482	2.732	0.021

**Table 12:** Model summary (mixed breastfeeding with low-risk pregnancy).

Model	*R*	*R* Square	Adjusted *R* Square	Std. Error of the Estimate
8	0.590	0.348	0.307	0.42823

**Table 13:** ANOVA (mixed breastfeeding with low-risk pregnancy).

Model		Sum of Squares	*df*	Mean Square	*F*	Sig.
8	Regression	1.566	1	1.566	8.539	0.010
	Residual	2.934	16	0.183		
	Total	4.500	17			

**Table 14:** Coefficients (mixed breastfeeding with low-risk pregnancy).

Model		Unstandardized Coefficients		Standardized Coefficients	*t*	Sig.
		*B*	Std. Error	Beta		
8	(Constant)	−1.430	1.008		−1.419	0.175
	Birth weight	0.001	0.000	0.590	2.922	0.010

## Data Availability

Data are contained within the article.
